# The Effect of FcγRIIIA Gene Polymorphism on the Treatment of Diffuse Large B-cell Non-Hodgkin Lymphoma: A Multicenter Prospective Observational Study

**DOI:** 10.4274/tjh.2013.0367

**Published:** 2015-05-08

**Authors:** Nurhilal Büyükkurt, Mehmet Ali Özcan, Ülkü Ergene, Bahriye Payzın, Sunay Tunalı, Fatih Demirkan, Hayri Özsan, Özden Pişkin, Bülent Ündar

**Affiliations:** 1 Başkent University Faculty of Medicine, Adana Education and Research Centre, Clinic of Hematology, Adana, Turkey; 2 Dokuz Eylül University Faculty of Medicine, Department of Hematology, İzmir, Turkey; 3 Celal Bayar University Faculty of Medicine, Department of Hematology, Manisa, Turkey; 4 Atatürk Training and Research Hospital, Clinic of Hematology, İzmir, Turkey

**Keywords:** FcγRIIIA, Diffuse large B-cell lymphoma, Rituximab

## Abstract

**Objective:**

The curative treatment approach for diffuse large B-cell lymphoma (DLBCL) is controversial even in the rituximab (R) era. The aim of this study was to examine the FcγRIIIA gene polymorphism distribution of DLBCL patients who had been treated with R-CHOP (cyclophosphamide, doxorubicin, vincristine, and prednisone) chemotherapy. Furthermore, we investigated the impact of FcγRIIIA gene polymorphism on the overall response rate (ORR) and overall survival (OS).

**Materials and Methods:**

Patients from 3 centers in the Aegean region of Turkey who had newly diagnosed CD20-positive DLBCL were enrolled in the study. The single nucleotide polymorphisms of the FcγRIIIA gene were analyzed by real time-PCR. The response to treatment was determined in the middle and at the end of the protocol. During 2 years of follow-up, the patients were clinically and radiologically evaluated for disease status every 3 months.

**Results:**

Thirty-six patients were included in the study and the distributions of F/F, V/F, and V/V types of alleles of FcγRIIIA were 25%, 50%, and 25%, respectively. Twenty-seven patients were considered as evaluable according to ORR and OS. The patients’ ORR was 87.5%, 100%, and 50% in the F/F, V/F, and V/V allele groups, respectively. We did not establish any statistically significant differences among the 3 alleles groups in respect to ORR (p=0.93). The OS within 2 years in the F/F, V/F, and V/V allele groups was 62.5%, 100%, and 100%, respectively. The OS in the F/F allele group was found to be lower than in the other 2 allele groups (p=0.01).

**Conclusion:**

The distribution of gene polymorphisms in our study group was similar to those of previous studies. While ORR was similar between the groups, our results highlight a lower OS in F/F patients compared to other allele groups of FcγRIIIA.

## Abstract (tr)

**Amaç:**

Diffüz büyük B hücreli non-Hodgkin lenfomada (DLBCL) kür sağlayıcı tedavi yaklaşımı rituximab çağında olmamıza rağmen tartışmalı bir konudur. Bu çalışmanın amacı R-CHOP (siklofosfamid, doksorubisin, vinkristin ve prednizon) rejimi alan DLBCL hastalarında FcγRIIIA gen polimorfizminin dağılımını incelemekti. Ayrıca FcγRIIIA gen polimorfizminin tüm yanıt oranları (ORR) ve tüm yaşam (OS) üzerine olan etkisini araştırmaktı.

**Gereç ve Yöntemler:**

Türkiye’nin Ege Bölgesi’ndeki üç merkezden yeni tanı almış CD-20 pozitif DLBCL hastaları çalışmaya dahil edildi. FcγRIIIA’daki tek gen polimorfizmi gerçek zamanlı-PCR ile incelendi. Tedaviye yanıt, planlanmış olan protokolün ortasında ve sonunda değerlendirildi. İki yıllık takip süresince her üç ayda bir hastalığın hem klinik, hem de radyolojik durumu ele alındı.

**Bulgular:**

Çalışmaya dahil edilen 36 hastada, FcγRIIIA’nın F/F, V/F ve V/V alellerinin dağılımı sırasıyla %25, %50 ve %25’ti. ORR ve OS verilerine göre 27 hasta değerlendirilebilir olarak kabul edildi. Hastaların ORR değerleri F/F, V/F ve V/V alel gruplarına göre sırasıyla %87,5; %100 ve %50 olarak hesaplandı. Hastaların ORR değerleri açısından üç alel grubu arasında istatistiksel olarak anlamlı fark saptanmadı (p=0,93). F/F, V/F ve V/V gruplarında iki yıllık OS %62,5, %100 ve %100 bulundu. F/F alel grubunun OS’si diğer iki alel grubundakinden daha düşük bulunmuştur (p=0,01).

**Sonuç:**

Gen polimorfizmi dağılımı sonuçlarımız önceki çalışmalarda bulunanlarla benzerdir. Gruplar arasında ORR değerleri arasında fark yokken, sonuçlarımız F/F hastalarının FcγRIIIA’nın diğer allel gruplarına göre daha kısa bir OS değerine sahip olduğunu göstermektedir.

## INTRODUCTION

Diffuse large B-cell lymphoma (DLBCL) is the most common histological subtype of non-Hodgkin lymphoma (NHL). It constitutes 25%-30% of NHLs [1,2]. Cyclophosphamide, doxorubicin, vincristine, and prednisone (CHOP) combined with rituximab (R) is the standard treatment protocol for DLBCL. Before the introduction of rituximab, which is a human-mouse chimerical anti-CD20 monoclonal antibody, CHOP was used alone. However, it was demonstrated that the addition of rituximab to the treatment protocol improves the complete remission rate and the 5-year event-free survival rate [3]. On the other hand, several studies have shown that R-CHOP has some limitations due to tumor pathobiology. 

The biological pathway of rituximab in the treatment of lymphomas is still controversial. According to the results of in vivo and in vitro studies, researchers have focused on 2 mechanisms: it increases the efficacy by inducing antibody-dependent cellular cytotoxicity (ADCC) and complement-dependent cytotoxicity (CDC) [4,5]. Natural killer (NK) cells, macrophages, and neutrophils play major roles in ADCC. When they recognize the constant region of the Fcγ receptors (FcγR) on the surface of immunoglobulin (Ig), they activate in order to initiate the ADCC cascade [6,7]. Macrophages, NK cells, and some dendritic cells express FcγRIIIA [8]. There may be valine (V) or phenylalanine (F) at the 158th position on the FcγRIIIA gene. An in vitro study showed that these gene polymorphisms (V/V, F/F, and V/F) change the binding affinities to immunoglobulin G (IgG). V/V alleles have the strongest affinity to IgG, whereas the F/F alleles have the lowest [9]. 

In the current study, we evaluated the distribution of FcγRIIIA gene single nucleotide polymorphism (SNP) in Turkish patients with DLBCL. The response rate to R-CHOP and overall survival (OS), regarding gene polymorphism, were also investigated.

## MATERIALS AND METHODS

### Patients’ Characteristics and Treatment Protocol

Thirty-six newly diagnosed DLBCL patients were included. The subjects were recruited from 3 hospitals in the Aegean region of Turkey over the course of 30 months. Immunohistochemistry staining was performed for all and the presence or absence of CD20 was examined. This study was approved by the Dokuz Eylül University Faculty of Medicine Hospital Ethics Committee and all patients signed an informed consent form.

The International Prognostic Index (IPI) score was calculated for the prediction of prognosis at the beginning of therapy. The chemotherapy regimen was administered in 3-week intervals. On the first day, rituximab 375 mg/m2 was given by intravenous infusion over 4-6 h. On the second day, cyclophosphamide 750 mg/m2, doxorubicin 50 mg/m2, and vincristine 1.4 mg/m2 (upper limit of 2 mg) were given intravenously, while prednisone 100 mg/m2 was administered orally on the second day and was then continued for 5 days. While stage 1 or 2 disease was generally treated with 4 cycles of R-CHOP followed by involved field radiotherapy (RT), the advanced stages of disease were treated with 6 to 8 cycles of chemotherapy followed by RT if there were bulky tumors. In order to assess the response to treatment, patients were evaluated after the second or fourth cycles of R-CHOP and also after all the planned cycles were completed. Monitoring and reevaluations of patients were performed every 3 months. All evaluations were performed according to the criteria of the International Lymphoma Workshop [10]. Patients fulfilling these follow-up criteria were classified as “evaluable”.

### FcγRIIIA Gene Polymorphism

Four milliliters of peripheral blood was collected into a tube containing EDTA. The genomic DNA was extracted and stored at -80 °C. The FcγRIIIA gene V158F polymorphism was determined by melting curve analysis after fluorescent real-time polymerase chain reaction (RT-PCR) on a Light Cycler (Roche Diagnostics, Basel, Switzerland). The RT-PCR was performed with the FcγRIIIA V158F Toolset for Light Cycler containing specific primers and fluorescent oligonucleotide probes and the Fast Start DNA Master Hybridization Probe Kit (Genes-4U, Neftenbach, Switzerland) according to the manufacturer’s instructions. The RT-PCR protocol consisted of an initial denaturation step at 95 °C for 2 min, followed by 40 cycles of 95 °C for 10 s, 56 °C for 10 s, and 72 °C for 10 s. The melting protocol consisted of a waiting process at 95 °C for 30 s and at 70 °C for 1 min, and a continuous fluorescence reading from 70 to 99 °C with a rising rate of 0.1 °C per second.

### Statistical Analysis

All available data were analyzed with SPSS 15. We performed descriptive analysis for patients’ age, sex, disease stage, extranodal involvement, bone marrow infiltration, and IPI score according to the FcγRIIIA gene allele groups. The variations of the clinical characteristics and treatment outcomes of the patients among the gene allele groups were compared with Kruskal-Wallis and Mann-Whitney U tests. The survival estimates were calculated by Kaplan-Meier technique and the differences of OS in gene allele groups were analyzed by log-rank test. All the analysis results were interpreted as statistically significant if the p-value was smaller than 0.05.

## RESULTS

There were 16 males (44.4%) and 20 females (55.6%). The median age was 61 (24-82) years at the time of diagnosis. The majority of patients (72.2%) had advanced stage disease (stages 3 and 4). Extranodal involvement was detected in 44.4% of the patients and B symptoms were present in 38.9%. High-intermediate and high IPI scores were observed in 36.1% (n=13) and 27.8% (n=10) of cases, respectively. One-fourth of patients had bulky lesions, and bone marrow infiltration was seen in 13.9%. The data concerning the patients’ characteristics in regard to FcγRIIIA gene alleles is exhibited in Table 1.

The V/F allele was the most frequent type of FcγRIIIA gene (50%). The incidences of the remaining alleles were equal (25%). We did not find any statistically significant differences among gene allele groups in terms of age, sex, B symptoms, extranodal involvement, bone marrow infiltration, stage, or IPI score (p=0.94, p=0.72, p=0.5, p=0.17, p=0.89, p=0.46, p=0.22, respectively). The overall response rate (ORR) to R-CHOP was examined in 27 patients. The rest could not be reached due to various reasons such as dying before the termination of protocol or moving to another city. 

According to our results, there was no statistically significant difference in the response rate among the 3 allele groups (p=0.93). We performed survival analysis at the end of the 30th month. Eight patients in the F/F allele group were considered as evaluable. Three of them died within the first year of therapy. One of them had progressive disease after R-CHOP and received salvage therapy. Fifteen patients in the V/F allele group were evaluable and all were alive. Four of 9 patients were evaluable in the V/V allele group and none of them died during the study interval.

**Figure 1:**
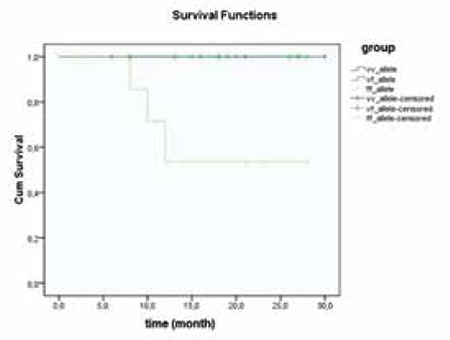
Overall survival curve of patients in each genotype.

The OS rate within 2 years was 62.5% for the F/F, 100% for the V/F, and 100% for the V/V allele group. We found statistically significant differences among the V/V-V/F and F/F allele groups for OS (p=0.01), as presented in Figure 1.

## DISCUSSION

Until the early 2000s, the standard therapy for DLBCL was the CHOP combination [11]. Since then, rituximab has been added, which was the first monoclonal antibody therapy approved by the Food and Drug Administration [12]. While significant improvement was achieved with rituximab, its mechanism of action is not clearly understood. In vitro and in vivo studies highlighted the importance of ADCC, CDC, and possibly the activation of the intracellular apoptosis signal pathway [4,5]. Recent studies showed that patients with follicular lymphoma, Waldenström’s macroglobulinemia, and chronic lymphocytic leukemia responded to rituximab treatment at different rates due to FcγRIIIA gene SNPs [13,14,15,16]. In 2006, Kim et al. stated that the estimated benefit of R-CHOP therapy in patients having DLBCL with the V/V allele is higher compared to patients with other allele types [8]. Ansell et al. demonstrated that rituximab improves the response rate not only when combined with chemotherapeutic agents, but also when combined with cytokines such as interleukin-12. These cytokines play a role in cellular cytotoxicity [17]. This report supported the idea that rituximab employs its anti-lymphoma effect by inducing major mediator cells of the ADCC pathway such as macrophages, NK cells, and dendritic cells. In this process, rituximab stimulates the effector cells of ADCC, which express FcγR after binding to CD20-positive B-cell lymphoma cells. FcγRIIIA is one of several types of Fcγ receptors. FcγRIIIA gene polymorphisms due to a point mutation at the 158th amino acid position may influence the responses of rituximab in patients with DLBCL. In their in vitro study, Hatjiharissi et al. indicated that individuals with FcγRIIIA V/V and V/F alleles showed higher rates of ADCC activity, because the NK cell surface has increased expression of FcγRIIIA, which leads to a remarkable affinity to rituximab [9]. 

Four previously published reports showed the clinical meaning of FcγRIIIA SNPs in DLBCL patients. In the first study, Kim et al. found a rapid response to R-CHOP in patients with the V/V allele in DLBCL compared to others allele groups [8]. The second was published by Zhang et al. They reported that V/V and V/F types of FcγRIIIA were evidently more responsive to initial R-CHOP therapy, as well as associated with longer survival [18]. The last 2 publications were made by Váróczy et al. and Mitroviç et al. [11,19]. A summary of these recent papers is depicted in Table 2. The results of the studies stated above are partially compatible with ours. They found no significance between the V/V and F/F alleles in term of event-free survival, OS, and ORR in R-CHOP treatment. As demonstrated in the last 2 papers, we did not find statistically significant differences in ORR, although OS was found significantly lower in F/F homozygous patients compared to the other 2 allele groups. According to this literature, there are 2 opposite observations on the same subject. We suppose that the reason for such contradicting results may be the different actions of the mechanism of rituximab other than the ADCC pathway, as well as different tumor pathobiologies. 

Our data have some limitations due to small sample size. To our knowledge, there are no other data available about this issue for Turkish patients. We suggest that these findings be assessed as preliminary data for Turkey. Our results were interpreted without drawing a precise conclusion.

## Figures and Tables

**Table 1:**
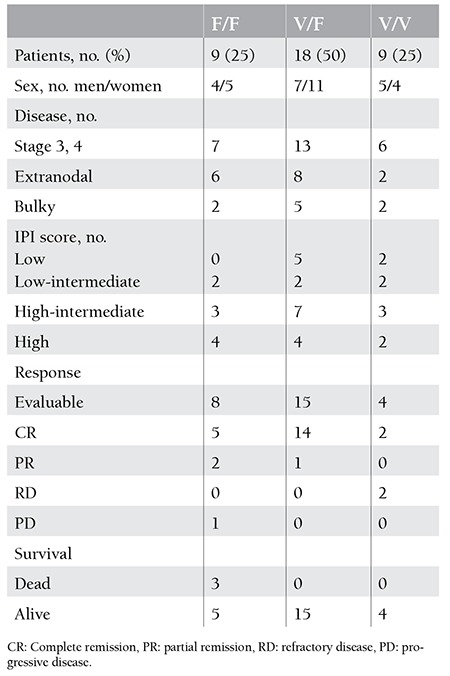
Patients’ characteristics and treatment outcomes with R-CHOP (cyclophosphamide, doxorubicin, vincristine, and prednisone) regimen according to FcγRIIIA gene alleles.

**Table 2:**
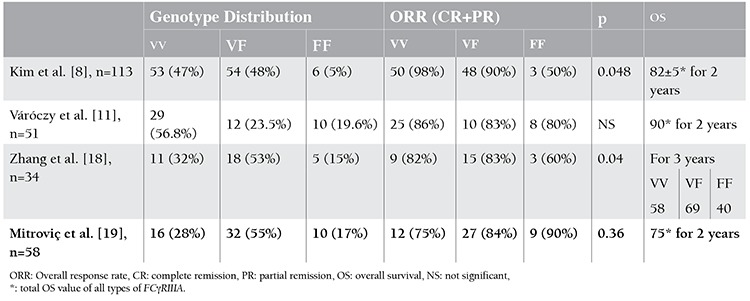
Summary of recent papers investigating the clinical importance of FCγRIIIA single nucleotide polymorphism (SNPs) in diffuse large B-cell lymphoma (DLBCL) patients.
